# Medical Journal Advertising

**Published:** 1892-10

**Authors:** 


MEDICAL JOURNAL ADVERTISING.—Hummel & Parmele, the indefatigable advertising agents and publishers of Philadelphia, have issued a very neat little book on this subject for the use of manufacturers and other advertisers. It contains many arguments to show the advantage of advertising in medical journals goods intended for physicians' use, and gives many points on the selection of a proper medium. The book also contains testimonials from successful advertisers, a variety of specimen advertisements, etc., and, in addition, it gives a classified list of the two hundred odd medical, surgical and sanitary journals published in the United States and Canada, together with their circulation—estimated by three directories, and in the last column, their own estimate. The book gives, also, many good reasons why a manufacturer should have his advertising business attended to by a live and conscientious agent, like Hummel & Parmele.

It is a very interesting and useful book, and all manufacturers of goods that doctors use should see it. The price is one dollar. Please see a notice on this subject elsewhere.

